# TIGIT-related transcriptome profile and its association with tumor immune microenvironment in breast cancer

**DOI:** 10.1042/BSR20204340

**Published:** 2021-03-24

**Authors:** Qin Zhang, Chaowei Gao, Jianqiang Shao, Zunyi Wang

**Affiliations:** 1 Thyroid and Breast Department III, Cangzhou Central Hospital, Cangzhou 061001, Hebei Province, China; 2 Breast Surgery Department, Chongqing University Three Gorges Hospital, Chongqing 404000, China

**Keywords:** Cancer immunotherapy, Immune response, Inflammatory activity, TIGIT

## Abstract

Immune checkpoints are intensively investigated as targets in cancer immunotherapy. T-cell immunoreceptor with immunoglobulin (Ig) and ITIM domains (TIGIT) are recently emerging as a novel promising target in cancer immunotherapy. Herein, we systematically investigated TIGIT-related transcriptome profile and relevant clinical information derived from a total of 2994 breast cancer patients recorded in The Cancer Genome Atlas (TCGA) and Molecular Taxonomy of Breast Cancer International Consortium (METABRIC). We uncovered the relationship between TIGIT and major molecular and clinical characteristics in breast cancer. More importantly, we depicted the landscape of associations between TIGIT and other immune cell populations. Gene ontology analyses and Gene Set Variation Analysis (GSVA) of genes correlated with TIGIT revealed that TIGIT were mainly involved in immune responses and inflammatory activities. In summary, TIGIT expression was tightly related to the aggressiveness of breast cancer; TIGIT might manipulate anti-tumor immune responses by impacting not only T cells but also other immune cells. To the best of our knowledge, this is by far the most comprehensive and largest study characterizing the molecular and clinical features of TIGIT in breast cancer through large-scale transcriptome data.

## Introduction

Breast cancer is the most frequently diagnosed cancers and the leading cause of cancer-related deaths in females worldwide [1]. One of the hallmarks of cancer is the escape of tumor cells from destruction induced by the immune system [2]. Induction of an exhausted phenotype in effector lymphocytes and thereby preventing effective tumor rejection represents one of the mechanisms of immune escape [3]. It is well accepted that several receptor/ligand systems are involved in inhibiting T-cell activation and thereby mediating checkpoint control of T-cell effector functions [4]. In recent years, cancer immunotherapy by inhibiting these immune checkpoints to reactivate the cytotoxic phenotype are emerging as a novel promising treatment strategy for cancer. Due to remarkable survival benefits of cancer immunotherapy, FDA approved the treatment of several cancer entities by using antibodies blocking the negative immune checkpoint molecules, cytotoxic T-lymphocyte-associated protein 4 (CTLA-4), programmed cell death protein 1 (PD-1) or PD-1 ligand 1 (PD-L1) [5]. However, a large proportion of cancer patients failed to respond to the treatment of blocking antibodies against the PD-1, PD-L1 or CTLA-4 immune checkpoint axis [6–8]. Hence, more endeavors are warranted to the therapeutic evaluation of additional immune checkpoints.

T-cell immunoreceptor with Ig and ITIM domains (TIGIT) is a type I transmembrane protein with an Ig-like variable extracellular domain expressed on activated and memory T cells, regulatory T cells, as well as natural killer (NK) and natural killer T cells (NKT) [9,10]. TIGIT was reported to be co-expressed with PD-1 on tumor antigen-specific CD8^+^ T cells and CD8^+^ tumor-infiltrating lymphocytes (TILs) in human cancer [10]. Moreover, co-expression of TIGIT and other inhibitory receptors on exhausted CD8^+^ T-cell subsets in tumors was also observed, such as T-cell immunoglobulin and mucin domain-containing molecule-3 (TIM-3) and lymphocyte activation gene 3 (LAG-3) [11]. Despite multiple lines of evidence support that TIGIT plays a pivotal role in limiting adaptive and innate immunity against tumors [4], the role of TIGIT and its association with tumor immune microenvironment in breast cancer remains largely unknown.

In the present investigation, a systematic analysis of large-scale TIGIT-related transcriptome profile was performed, which uncovered the potential role of TIGIT in mediating an immune response and inflammatory activities. To our knowledge this is the first integrative study characterizing the landscape of TIGIT expression in breast cancer both molecularly and clinically.

## Materials and methods

### Data collection

Transcriptome data of The Cancer Genome Atlas (TCGA) was downloaded and processed through GDCRNA Tools [12]. Raw counts data were normalized through TMM implemented in edgeR [13] and was then transformed by voom in limma [14], The edgeR and limma packages available from the Bioconductor project offer a well-developed suite of statistical methods for dealing with this question for RNA-seq data, and only genes with cpm > 1 in more than half of the samples were kept. Standardized survival data of TCGA cohort were downloaded from TCGA Pan-Cancer Clinical Data Resource (TCGA-CDR) [15]. Molecular Taxonomy of Breast Cancer International Consortium (METABRIC) dataset [16] containing a total of 1904 tumor cases were downloaded from cBioPortal database (http://www.cbioportal.org/).

### Bioinformatics analyses and statistical analyses

TIGIT expression pattern in human cancer was analyzed using the TIMER database [17], gene ontology analyses of the genes correlated with TIGIT were performed using clusterProfiler package [18]. Immune-related genes were retrieved from The Immunology Database and Analysis Portal (ImmPort) database (https://www.immport.org/home) [19]. Genes correlated with TIGIT expression was selected through batch computing the correlation coefficent between TIGIT and all genes. The absolute abundance of immune cell populations were calculated using a bioconductor package named Microenvironment Cell Populations-counter algorithm [20]. Gene Set Variation Analyses (GSVAs) [21] was perfomred to calculate the scores of gene sets correlated with immune functions and inflamatory activities [22]. Correlations between continuous variables were calculated by Pearson correlation method. Any differences in variables between groups were estimated through the Student’s *t* test, one-way ANOVA or Pearson’s Chi-squared test. All statistical tests were performed by using R language (version 3.6.3; https://www.r-project.org/). Other statistical calculations and graphical work were performed using several packages including ggplot2 [23], pheatmap, circlize [24], and corrgram [25]. *P*-value of less than 0.05 was considered statistically significant. All statistical tests were two-sided.

## Results

### TIGIT expression pattern in human cancer

**Figure 1:**
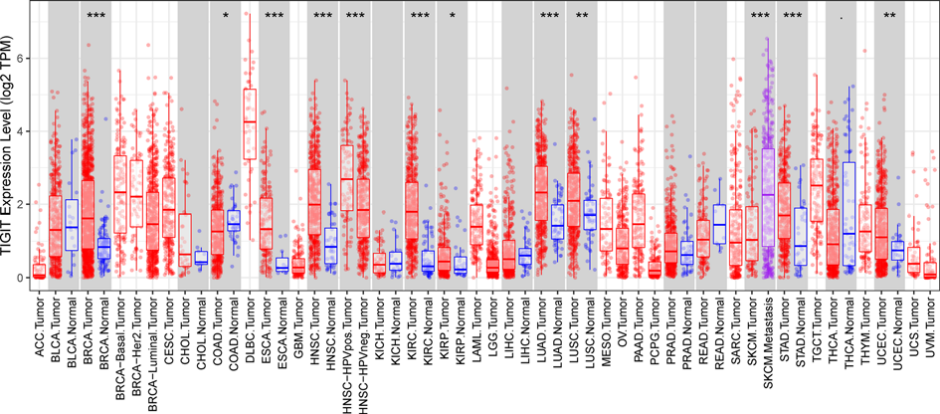
TIGIT expression status in pan-cancer TIGIT expression levels in all tumors and adjacent normal tissues across TCGA (*P<0.05, **P<0.01, ***P<0.001).

**Figure 2:**
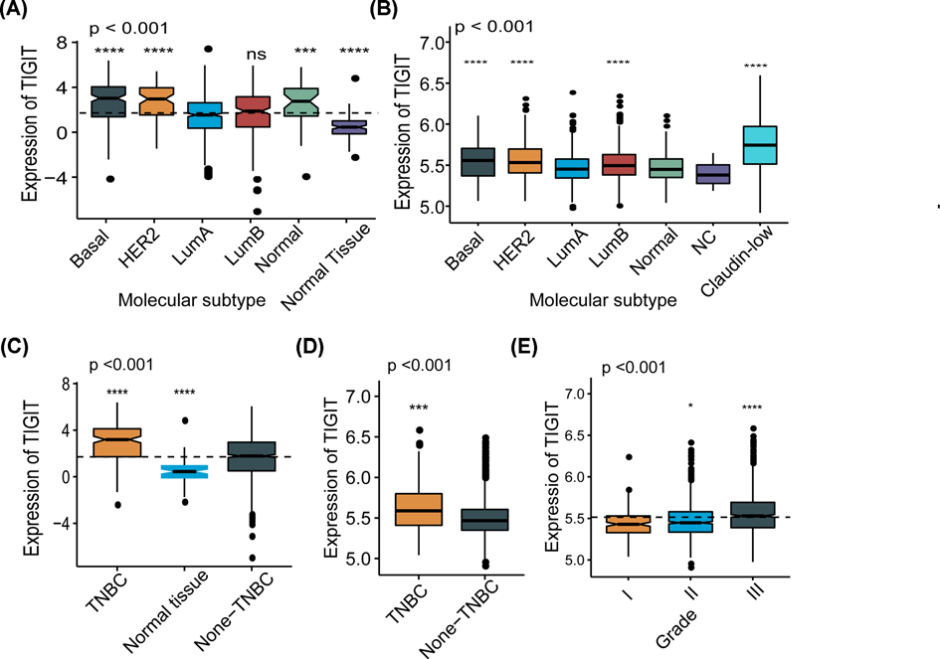
TIGIT expression in different molecular subtypes of transcriptional classification scheme in TCGA and METABRIC cohort (*P<0.05, ***P<0.001, ****P<0.0001). TIGIT expression pattern by molecular subtype (**A,B**), TIGIT expression pattern by TNBC status (**C,D**), TIGIT expression by tumor grade (**E**).

The expression of TIGIT in tumor and adjacent normal tissues across all tumors in TCGA were shown in Figure 1. Intriguingly, we found TIGIT was significantly up-regulated in the majority of the tumors when compared with normal tissues, but not BLCA (urothelial bladder carcinoma), CHOL (cholangiocarcinoma), COAD (colon adenocarcinoma), KICH (kidney chromophobe), LIHC (liver hepatocellular carcinoma), READ (rectum adenocarcinoma), and THCA (thyroid carcinoma). To further characterize the expression pattern of TIGIT in breast cancer, then we examined the RNA-sequencing data of breast cancer from TCGA and METABRIC databases, the association of TIGIT and clinical characteristics are listed in Tables 1 and 2. Compared with LuminalA subtype, Basal-like and HER2-enriched subtype showed significant higher expression in TCGA cohort (*n*=1090), and the sample counts information is supplied in Supplementary Table S1 (Figure 2A), and most of the result and the global tendency were well-validated in METABRIC cohort (*n*=1994), considering the context that the subtype category was not exactly the same with each other in these two databases (Figure 2B). Moreover, TIGIT overexpression was also observed in triple-negative breast cancer (TNBC) than none-TNBC samples (Figure 2C,D). TIGIT also showed higher expression in higher tumor grade (Figure 2E). These results suggested that TIGIT was highly specifically expressed in more malignant breast cancer. Based on these findings, we inferred that TIGIT may play important biologic functions in breast cancer.

**Table 1:** Association between TIGIT mRNA expression and clinicopathologic characteristics in TCGA cohort

	Total (*n*=1090)	Expression		*P*-value
		TIGIT high (*n*=545)	TIGIT low (*n*=545)	
**Age (years)**				
≥55	517 (47.4%)	273 (50.1%)	244 (44.8%)	0.089
<55	573 (52.6%)	272 (49.9%)	301 (55.2%)	
**T stage**				
T1	279 (25.6%)	132 (24.2%)	147 (27.0%)	0.003
T2	631 (57.9%)	340 (62.4%)	291 (53.4%)	
T3	137 (12.6%)	62 (11.4%)	75 (13.8%)	
T4	40 (3.7%)	11 (2.0%)	29 (5.3%)	
Unknown	3 (0.3%)	0 (0%)	3 (0.6%)	
**N stage**				
N0	514 (47.2%)	254 (46.6%)	260 (47.7%)	0.372
N1	360 (33.0%)	176 (32.3%)	184 (33.8%)	
N2	120 (11.0%)	68 (12.5%)	52 (9.5%)	
N3	76 (7.0%)	42 (7.7%)	34 (6.2%)	
Unknown	20 (1.8%)	5 (0.9%)	15 (2.8%)	
**M stage**				
M0	907 (83.2%)	460 (84.4%)	447 (82.0%)	0.345
M1	22 (2.0%)	8 (1.5%)	14 (2.6%)	
Unknown	161 (14.8%)	77 (14.1%)	84 (15.4%)	
**AJCC stage**				
I	181 (16.6%)	81 (14.9%)	100 (18.3%)	0.201
II	621 (57.0%)	320 (58.7%)	301 (55.2%)	
III	250 (22.9%)	129 (23.7%)	121 (22.2%)	
IV	20 (1.8%)	7 (1.3%)	13 (2.4%)	
Unknown	18 (1.7%)	8 (1.5%)	10 (1.8%)	
**ER status**				
Negative	236 (21.7%)	168 (30.8%)	68 (12.5%)	<0.001
Positive	803 (73.7%)	357 (65.5%)	446 (81.8%)	
Unknown	51 (4.7%)	20 (3.7%)	31 (5.7%)	
**PR status**				
Negative	343 (31.5%)	211 (38.7%)	132 (24.2%)	<0.001
Positive	694 (63.7%)	313 (57.4%)	381 (69.9%)	
Unknown	53 (4.9%)	21 (3.9%)	32 (5.9%)	
**HER2 status**				
Negative	895 (82.1%)	440 (80.7%)	455 (83.5%)	0.004
Positive	168 (15.4%)	98 (18.0%)	70 (12.8%)	
Unknown	27 (2.5%)	7 (1.3%)	20 (3.7%)	

**Table 2:** Association between TIGIT mRNA expression and clinicopathologic characteristics in METABRIC cohort

	Total (*n*=1904)	Expression		*P*-value
		TIGIT high (*n*=952)	TIGIT low (*n*=952)	
**Age (years)**				
≥55	952 (50.0%)	494 (51.9%)	458 (48.1%)	0.109
<55	952 (50.0%)	458 (48.1%)	494 (51.9%)	
**Tumor size**				
≥2 cm	592 (31.1%)	294 (30.9%)	298 (31.3%)	0.956
<2 cm	1292 (67.9%)	645 (67.8%)	647 (68.0%)	
Unknown	20 (1.1%)	13 (1.4%)	7 (0.7%)	
**AJCC stage**				
0	4 (0.2%)	3 (0.3%)	1 (0.1%)	0.288
I	475 (24.9%)	237 (24.9%)	238 (25.0%)	
II	800 (42.0%)	382 (40.1%)	418 (43.9%)	
III	115 (6.0%)	66 (6.9%)	49 (5.1%)	
IV	9 (0.5%)	4 (0.4%)	5 (0.5%)	
Unknown	501 (26.3%)	260 (27.3%)	241 (25.3%)	
**Tumor Grade**				
I	165 (8.7%)	62 (6.5%)	103 (10.8%)	<0.001
II	740 (38.9%)	317 (33.3%)	423 (44.4%)	
III	927 (48.7%)	544 (57.1%)	383 (40.2%)	
Unknown	72 (3.8%)	29 (3.0%)	43 (4.5%)	
**ER status**				
Negative	445 (23.4%)	294 (30.9%)	151 (15.9%)	<0.001
Positive	1459 (76.6%)	658 (69.1%)	801 (84.1%)	
**PR status**				
Negative	895 (47.0%)	536 (56.3%)	359 (37.7%)	<0.001
Positive	1009 (53.0%)	416 (43.7%)	593 (62.3%)	
**HER2 status**				
Negative	1668 (87.6%)	808 (84.9%)	860 (90.3%)	<0.001
Positive	236 (12.4%)	144 (15.1%)	92 (9.7%)	

### TIGIT-related biological process

**Figure 3:**
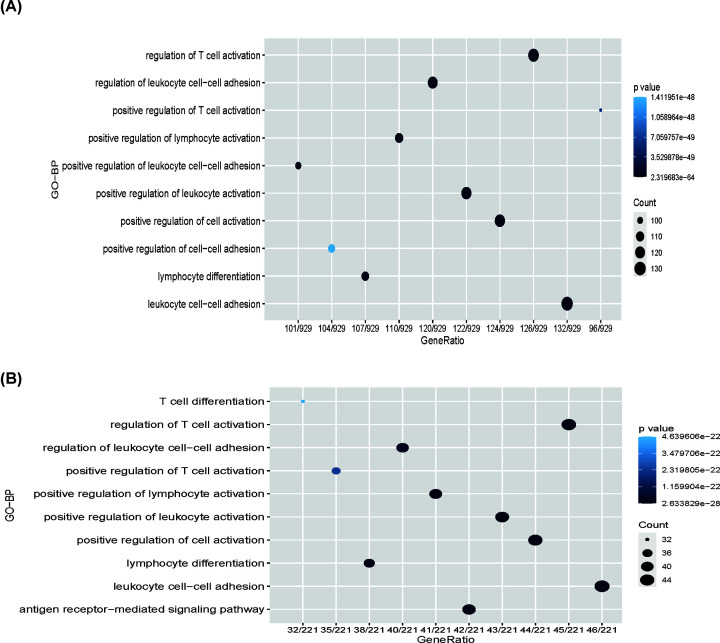
TIGIT was closely related to immune functions in breast cancer Gene ontology analysis showed that TIGIT was mainly involved in immune response and inflammatory response in TCGA and METABRIC cohort (**A,B**).

To further characterize the potential biological functions of TIGIT, genes correlated with TIGIT expression (Pearson |R| ≥ 0.4) were selected out (*n*=1010) (Supplementary Table S2), these genes were further used to perform functional enrichment analyses using clusterProfiler package in R [18]. Interestingly, when the gene function was sorted by *P*-value in increasing order, TIGIT-related genes were mainly enriched in immune and inflammation-related biological processes, including pathways relevant to regulation of T-cell activation, leukocyte cell–cell adhesion, and regulation of leukocyte cell activation (Figure 3A,B). Of note, these results can be mutually validated in TCGA and METABRIC at a global level. To further explore the immune function of TIGIT in specific immune functions, we then investigated the role of the specific immune roles of TIGIT in immune response and inflammatory activities.

### TIGIT-related immune response

**Figure 4:**
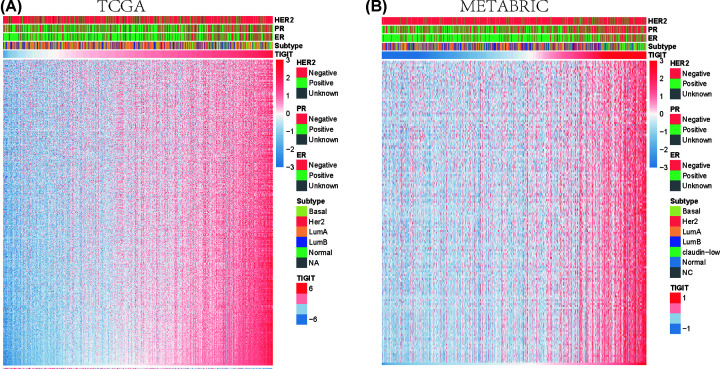
TIGIT-related immune responses Most immune-related genes are positively correlated with TIGIT expression in TCGA and METABRIC databases, while few genes are negatively associated (**A,B**). The bar followed with TIGIT represents the expression of TIGIT from low to high. The x-axis of heatmap represents the samples ordered by the expression of TIGIT, and y-axis represents the immune-related genes. The color of map represents the normalized expression value of genes.

To further characterize the biological role of TIGIT in the immune response in breast cancer, a total of 4723 immune-related genes were downloaded from The Immunology Database and Analysis Portal (ImmPort) database mj. We selected the genes that were most relevant to TIGIT (Pearson |R| > 0.4, *P*<0.05) to depict the correlation pattern of TIGIT and immune-related genes. Among the genes, 507 and 158 were positively correlated with TIGIT expression in TCGA and METABRIC, respectively, while the number of negatively related genes was 1 and 0, respectively (Figure 4A,B), the gene lists were supplied as Supplementary Table S3. These results suggest that TIGIT is positively correlated with most relevant immune responses and negatively correlated with few immune responses in breast cancer, indicating the important association of TIGIT and immune response.

### Association of TIGIT expression and immune cell populations

**Figure 5:**
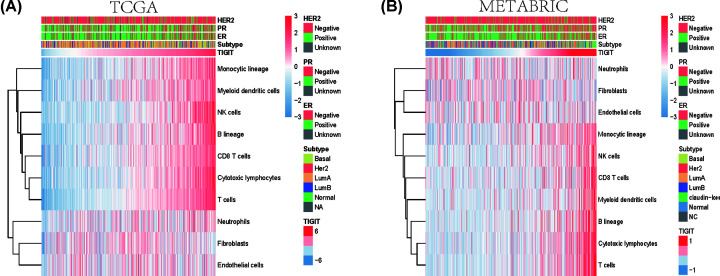
Association between TIGIT expression and immune cell populations in TCGA and METABRIC cohort The bar followed with TIGIT represents the expression of TIGIT from low to high. The x-axis of heatmap represents the samples ordered by the expression of TIGIT, and y-axis represents immune cells. The color of map represents the score of immune cells (**A,B**).

To further uncover the functional role of TIGIT in breast cancer immune microenvironment, the absolute abundance of eight immune and two stromal cell populations was estimated from the transcriptome data using the Microenvironment Cell Populations-counter method developed by Becht et al. [20]. TIGIT expression was found to be positively correlated with T cells, CD8 T cells, Cytotoxic lymphocytes, NK cells, B lineage, Monocytic lineage and Myeloid dendritic cells, but not with Neutrophils, Endothelial cells and Fibroblasts, this pattern was consistent in both TCGA and METABRIC cohort at a global level (Figure 5A,B). These findings suggest that TIGIT might not only involved in regulating T-cell immunity, but also other immune cells immunity, including Cytotoxic lymphocytes, NK cells, B lineage, Monocytic lineage and Myeloid dendritic cells. Of note, these results can also be mutually validated in TCGA and METABRIC, which make our results more reliable.

### Association of TIGIT expression and inflammatory activities

**Figure 6:**
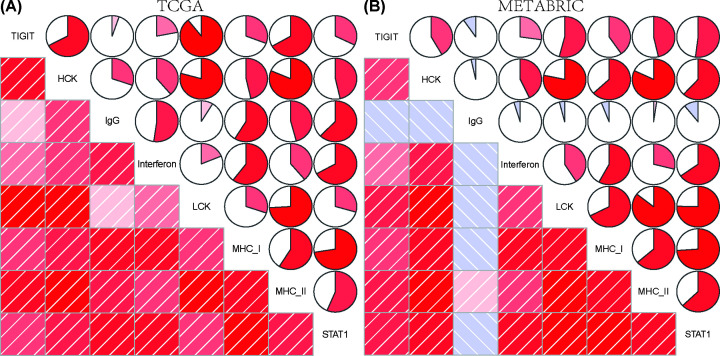
The relationship between TIGIT expression and inflammatory activities in TCGA and METABRIC cohort The percentage size of pie represents the correlation coefficients between TIGIT and related metagenes, larger percentage size represents higher coefficient. The redder the color, the higher the coefficients between TIGIT and metagenes (**A,B**).

To further investigate the role of TIGIT-related inflammatory activities, seven clusters of 104 genes in total were subsequently defined as metagenes [26] (Supplementary Table S4), representing different types of inflammation and immune response. Intriguingly, we found TIGIT expression was positively correlated with LCK, HCK, MHC-I, MHC-II, STAT1 and interferon in both TCGA and METABRIC databases (Figure 6A,B). These results implicated that TIGIT was involved in the activating of macrophages, T cells signaling transduction, and antigen-presenting cells. However, the association between TIGIT expression and IgG showed inconsistent results in two independent databases, thereby suggesting the role of TIGIT in regulating B linage-related immune responses is still not clear. These findings are consistent with the above results and further confirmed the important biological functions of TIGIT in breast cancer immune microenvironment.

## Discussion

Breast cancer is one of the leading causes that affects human women’s health severely. Therefore, new therapeutic options are urgently needed. Recently, immunotherapy has shown a promising prospect for breast cancer patients, especially for TNBC. In the past few decades, studies in the field of cancer immunotherapy have been mainly focused on CTLA-4 and PD-1/PD-L1 blockade, However, since the complex interactions between tumor immune modulators, targeting PD-1/PDL-1 alone might be insufficient. Multiple lines of evidence support that TIGIT might be an intriguing candidate for immunotherapy of breast cancer in the future [4,10,11,27].

In the past few decades, most of studies have been focused on the role of PD-1/PDL-1, and CTLA-4 in breast cancer. Only very limited studies pay attention to TIGIT. The ligand of TIGIT, poliovirus receptor (PVR) was reported to be associated with more aggressive breast cancer subtypes such as HER2 positive and TNBC [28], which is consistent with our results. *In vitro* evidence support that blocking TIGIT or PVR resulted in enhanced immune cell-mediated lysis of breast cancer cell lines [28].

Herein, we analyzed the TIGIT expression in the RNA-seq data of 2994 breast cancer patients. As expected, TIGIT expression was significantly up-regulated in higher malignant pathological type breast cancer. Moreover, we also found that high expression of TIGIT was highly enriched in more aggressive breast cancer subtypes, including higher tumor grade, TNBC subtype, basal-like subtype and HER2-enriched subtype. It is most likely that these malignant biologic behaviors have contributed to tumor recurrence and therapy resistance, which implicates that mechanism of TIGIT in breast cancer might be the key to triumphing over this deadly disease. Interestingly, we found that TIGIT played almost exactly the same role in immune and inflammatory response as PD-1 in breast cancer [29]. Together, these data indicated that TIGIT and PD-1 might play a synergistic role in tumor immune and inflammatory response to promote the development of a severe dysfunctional phenotype in T cells in breast cancer as reported in other cancers [27,30].

In summary, TIGIT expression is closely related to the aggressiveness of breast cancer and might be a potential biomarker. PD-1 might manipulate anti-tumor immune response by impacting not only T cells but also other immune cells, and this might vary with different tumors. Furthermore, TIGIT might synergize with other immune checkpoint molecules to regulate the immune microenvironment in breast cancer, which shed novel sights for developing new targeted drugs for immunotherapy. To the best of our knowledge, this is by far the most comprehensive and largest study investigating the molecular and clinical features of TIGIT in breast cancer using large-scale transcriptome data. Our work further supports that TIGIT might be a promising target for breast cancer immunotherapy, future work focused on elaborating the potential synergistic role between TIGIT and other immune check point molecules will provide novel sights for combination immunotherapy.

## Supplementary Material

Supplementary Tables S1-S4

## Data Availability

The data generated or analyzed during the present study are included in this article, or if absent are available from the corresponding author upon reasonable request.

## Abbreviations

CTLA-4: cytotoxic T-lymphocyte-associated protein 4
Ig: immunoglobulin
METABRIC: Molecular Taxonomy of Breast Cancer International Consortium
NK: natural killer
PD-1: programmed cell death protein 1
PD-L1: PD-1 ligand 1
PVR: poliovirus receptor
TCGA: The Cancer Genome Atlas
TIGIT: T-cell immunoreceptor with Ig and ITIM domains
TNBC: triple-negative breast cancer
